# Unusual Presentation of Multi-organ Hydatid Cysts in a Child

**DOI:** 10.4274/balkanmedj.galenos.2019.2019.2.31

**Published:** 2019-08-22

**Authors:** İsa Cam, Özgür Çakır, Ayşe Tekin Yılmaz, Samet Genez, Yonca Anık

**Affiliations:** 1 Department of Radiology, Kocaeli University School of Medicine, Kocaeli, Turkey; 2 Department of Infectious Diseases and Clinical Microbiology, Kocaeli University School of Medicine, Kocaeli, Turkey; 3 Clinic of Radiology, Derince Training and Research Hospital, Kocaeli, Turkey

A 9-year-old boy presented to the pediatric clinic with symptoms of headache, left-sided diplopia, impaired speech, and nausea. His leukocyte count was 14100/μL with eosinophilia of 3050/μL (21.7%). Cranial magnetic resonance imaging revealed the presence of a cyst measuring 7×6 cm in the left parietal region with some mass effect over the adjacent structures and minimal perilesional edema. The lesion was solitary, homogeneous, and spherical with well-defined borders and no contrast enhancement (Figure 1a). Initial radiological diagnosis was that of hydatid disease. Subsequent thoracic radiography revealed multiple, well-delineated, round opacities (Figure 1b). Multiple cystic lesions were also detected on the liver upon abdominal magnetic resonance imaging and ultrasound imaging (Figure 1c). Echinococcal enzyme-linked immunosorbent assay as well as indirect hemagglutination and and hydatid cyst antibody tests showed positive results, confirming the diagnosis of hydatid disease. Oral albendazole treatment at 400 mg was initiated along with 4×3 mg of intravenous dexamethasone to treat perilesional edema associated with the brain lesion. After 7 days of presentation, the brain cyst was surgically removed without any complications. Pathology was consistent with that of a cerebral hydatid cyst.

Albendazole was continued at a dose of 10 mg/kg/day. After 56 months of treatment, follow-up images of the lesion sites were obtained. Brain magnetic resonance imaging revealed encephalomalacic changes at the surgical site; however, no recurrence of the hydatid cyst was detected (Figure 2a). Total regression in both the lungs (Figure 2b) and liver (Figure 2c) was observed with no recurrence of the hydatid cyst. Informed consent for publication was obtained from the patient’s family.

This case describes an extremely rare presentation of hydatid disease. Only 1% of hydatid disease cases have been reported to involve the central nervous system, with the lesions generally being adjacent to the middle cerebral artery (1). Hydatid disease accounts for only 1%-2% of pediatric cerebral space-occupying lesions (2), and the most common symptoms associated with primary intracranial cysts include headache, papilledema, diplopia, nausea, and vomiting, some of which were observed upon presentation of our case. Symptoms caused due to increased intracranial pressure may also manifest (2). Hydatid disease generally involves the liver (50%-70%) and less commonly the lungs (20%-30%); cysts have also been reported in other tissues, including the heart, genitourinary system, and soft and skeletal tissues. Hydatid disease typically demonstrates characteristic imaging findings (1,3). Additional diagnostic testing should include serological testing even though it has a variable sensitivity ranging 50%-98% (3,4). Therefore, negative serological tests cannot be used to rule out hydatid disease. In the present study, albendazole therapy was started because it has been suggested to reduce disease recurrence (2,3).

Diagnosing hydatid disease requires abdominal ultrasound imaging and chest radiography to investigate the liver and lung. Computed tomography and magnetic resonance imaging are useful techniques for diagnosing disseminated disease. Serological tests are helpful but are not diagnostic and cannot exclude hydatid disease. Management of disseminated hydatid disease is complex and requires a multidisciplinary approach. Albendazole therapy should be initiated because of its efficacy in preventing hydatid disease recurrence.

## Figures and Tables

**Figure 1:**
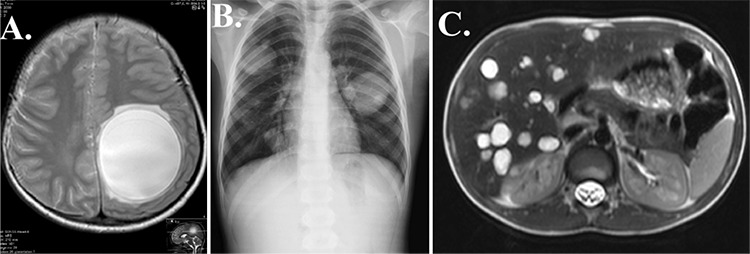
Images of the patient at presentation: Cranial magnetic resonance imaging T2-weighted axial image showing a large hydatid cyst with perilesional edema (a). Chest radiography showing multiple hydatid cysts (b). T2-weighted axial image at the level of the liver showing multiple hydatid cystic lesions (c).

**Figure 2:**
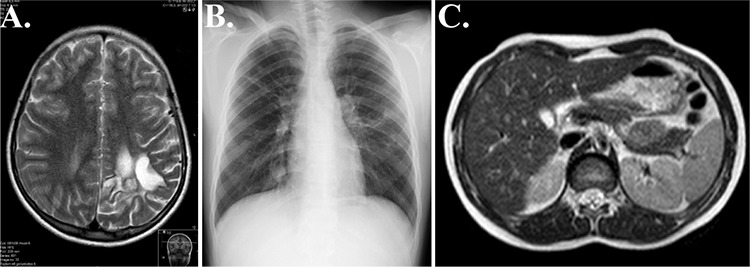
Repeat images after 56 months of albendazole therapy: Encephalomalacic changes were observed at the site of surgery on postoperative T2-weighted axial image (a). Chest radiography showing complete regression of hydatid cysts (b). T2-weighted axial image of the liver with total regression of hydatid cysts (c).
